# Monitoring the Progress towards the Elimination of Gambiense Human African Trypanosomiasis

**DOI:** 10.1371/journal.pntd.0003785

**Published:** 2015-06-09

**Authors:** Pere P. Simarro, Giuliano Cecchi, José R. Franco, Massimo Paone, Abdoulaye Diarra, Gerardo Priotto, Raffaele C. Mattioli, Jean G. Jannin

**Affiliations:** 1 World Health Organization, Control of Neglected Tropical Diseases, Innovative and Intensified Disease Management, Geneva, Switzerland; 2 Food and Agriculture Organization of the United Nations, Sub-regional Office for Eastern Africa, Addis Ababa, Ethiopia; 3 Food and Agriculture Organization of the United Nations, Animal Production and Health Division, Rome, Italy; 4 World Health Organization, Regional Office for Africa, Intercountry Support Team, Libreville, Gabon; Makerere University, UGANDA

## Abstract

**Background:**

Over the last few years, momentum has gathered around the feasibility and opportunity of eliminating gambiense human African trypanosomiasis (g-HAT). Under the leadership of the World Health Organization (WHO), a large coalition of stakeholders is now committed to achieving this goal. A roadmap has been laid out, and indicators and milestones have been defined to monitor the progress of the elimination of g-HAT as a public health problem by 2020. Subsequently, a more ambitious objective was set for 2030: to stop disease transmission. This paper provides a situational update to 2012 for a number of indicators of elimination: number of cases annually reported, geographic distribution of the disease and areas and populations at different levels of risk.

**Results:**

Comparing the 5-year periods 2003-2007 and 2008-2012, the area at high or very high risk of g-HAT shrank by 60%, while the area at moderate risk decreased by 22%. These are the areas where g-HAT is still to be considered a public health problem (i.e. > 1 HAT reported case per 10,000 people per annum). This contraction of at-risk areas corresponds to a reduction of 57% for the population at high or very high risk (from 4.1 to 1.8 million), and 20% for moderate risk (from 14.0 to 11.3 million).

**Discussion:**

Improved data completeness and accuracy of the Atlas of HAT enhanced our capacity to monitor the progress towards the elimination of g-HAT. The trends in the selected indicators suggest that, in recent years, progress has been steady and in line with the elimination goal laid out in the WHO roadmap on neglected tropical diseases.

## Author Summary

Control activities conducted over the last 15 years against gambiense human African trypanosomiasis (g-HAT) have had a tremendous impact on disease transmission, and the elimination of g-HAT now appears achievable. In this context, accurate monitoring is crucial. This paper analyzes g-HAT epidemiological trends by comparing two periods: 2003–2007 and 2008–2012. The number of reported cases decreased from 19,963 in 2003 to 7,106 in 2012. The areas at high or very high risk shrank by 60% between the two study periods. For 2008–2012, 43.4 million people out of a total of 56.4 million at risk lived in areas at low or very low risk of infection, and they have therefore met the criterion of elimination as a public health problem (i.e. < 1 case per 10,000 inhabitants per year). The challenge for the future is twofold. First, to prevent these 43.4 million people from sliding back into a situation of higher risk through effective surveillance. Second, to develop sustainable and adapted strategies to curb transmission in the areas where people are still living at moderate to very high risk. The WHO network for g-HAT elimination provides an opportunity to synergize efforts and to overcome the hurdles in this challenging endeavour.

## Introduction

Thanks to the efforts of a wide range of stakeholders, as well as to the commitment of countless field workers in affected countries, the elimination of gambiense human African trypanosomiasis (g-HAT) seems achievable.

In 2001, when the number of infected people was reaching alarming levels [1], the World Health Organization (WHO) and its partners launched a public-private partnership that, combined with the efforts of NGOs and bilateral cooperation, resulted in enhanced disease control and a sizable reduction in the number of cases. In 2003 and 2004, high-level political willingness led the World Health Assembly to pass resolutions (WHA56.7 and WHA57.2) that urged to strengthen disease control and to aim at elimination. In subsequent years, further progress was made in decreasing the number of reported cases, until in 2011 the WHO Strategic and Technical Advisory Group on Neglected Tropical Diseases (NTDs) judged that the elimination goal was achievable. A roadmap on NTDs was produced, which targets, among others, the elimination of HAT as a public health problem by 2020 [2].

Over the last three years momentum has kept building. On 30 January 2012, a diverse and committed gathering of actors from the public and private sectors aligned itself with WHO objectives and issued the ‘London Declaration on Neglected Tropical Diseases’. The Declaration focused on 10 infections that affect the world’s poorest populations, and HAT was targeted for elimination. A meeting organized by WHO in late 2012 with g-HAT-affected countries, cemented this commitment. A more ambitious goal was also set for 2030: the complete interruption of transmission [3].

In April 2013, a WHO Expert Committee on control and surveillance of HAT was convened [4]. The experts recognized the feasibility of g-HAT elimination, and they identified and endorsed two primary indicators to measure the progress towards elimination: (i) the number of cases annually reported, and (ii) the number of foci validated as eliminated (i.e. reporting less than 1 case per 10,000 inhabitants per annum—p.a.). Both indicators will be monitored annually. However, for the latter indicator, monitoring is planned to start in 2015 and, as such, it is not addressed in this paper any further.

Secondary indicators were also defined, which are intended to assess the intensity and effectiveness of the elimination activities. These include: (i) the geographical distribution of the disease, (ii) the areas and populations at different levels of risk, and (iii) the proportion of the population at risk covered by control and surveillance activities. The latter indicator is not discussed further in this paper as the coverage of passive surveillance has already been analyzed elsewhere [5], and the coverage of active surveillance will be the object of a separate, dedicated publication.

In the present paper, two 5-year periods are analyzed (i.e. 2003–2007 and 2008–2012), with a view towards assessing trends and measuring progress. The geographical extent of the disease and the areas and populations at risk herewith presented also provide an update of previously published estimates for the period 2000–2009 [6,7].

## Materials and Methods

### Number of cases annually reported

This indicator is based on annual figures reported to WHO by National Sleeping Sickness Control Programmes (NSSCPs) and NGOs. The tally is complemented by cases detected in non-endemic countries, concerning essentially travellers and migrants. These are linked to the location where they have most probably been infected [8].

### Gambiense HAT geographic distribution

The geographic distribution of g-HAT presented in this paper is based on the data contained in the database of the Atlas of HAT for the 10-year period 2003–2012 [4], for a total of 115,368 cases. Mapping was carried out using methodologies already described [6,9].

### Areas and population at risk

The risk of g-HAT infection was estimated using previously developed methods [7,10]. In a nutshell, risk is expressed as the ratio of two surfaces: disease intensity and population intensity. The former is based on g-HAT occurrence data as assembled in the Atlas of HAT, the latter relies on estimations of human population density as provided by Landscan databases [11]. Both intensity surfaces are calculated through kernel smoothing [12], by using a search radius of 30 km. On the basis of the number of HAT cases p.a., risk is subsequently categorized as ‘very low’ (≥ 1 per 10^6^ people and < 1 per 10^5^ people), low (≥ 1 per 10^5^ people and < 1 per 10^4^ people), moderate (≥ 1 per 10^4^ people and < 1 per 10^3^ people), high (≥ 1 per 10^3^ people and < 1 per 10^2^ people) and ‘very high’ (≥ 1 per 10^2^ people)[10]. Below the threshold of 1 HAT case per 1 million people p.a. risk is considered ‘marginal’. Importantly, risk categories below the level of ‘moderate’ (i.e. ‘low’ and ‘very low’) meet the objective set by WHO for the elimination as a public health problem.

While for previous studies disease distribution and risk were estimated for the 10-year period 2000–2009 [7,10], in the present paper we look at the period 2003–2012. This is because, as we write, the database of the Atlas of HAT provides consolidated data up to 2012. Data for 2013 onwards are still under processing and verification. The trend is explored by splitting the analysis into two 5-year periods (2003–2007 and 2008–2012). For both periods, the estimation of the risk layer is made by using as denominator the mean of the five years’ population (Landscan datasets), while the calculation of the population at risk, as a standalone indicator, is based on the Landscan population at the end of each period (i.e. 2007 and 2012 respectively) [7,10].

For the present run of risk estimation the Atlas of HAT provided village-level mapping for 92.1% of g-HAT reported cases. For the remaining 7.9% of the cases for which village-level information was not available, focus-level information was used. In particular, cases unmapped at the village-level but with known area of occurrence (e.g. focus, parish, health zone, etc.) were allocated proportionally to the endemic villages of the same area using methods already described [10].

## Results

### Trend in the number of gambiense HAT cases annually reported

**Fig 1:**
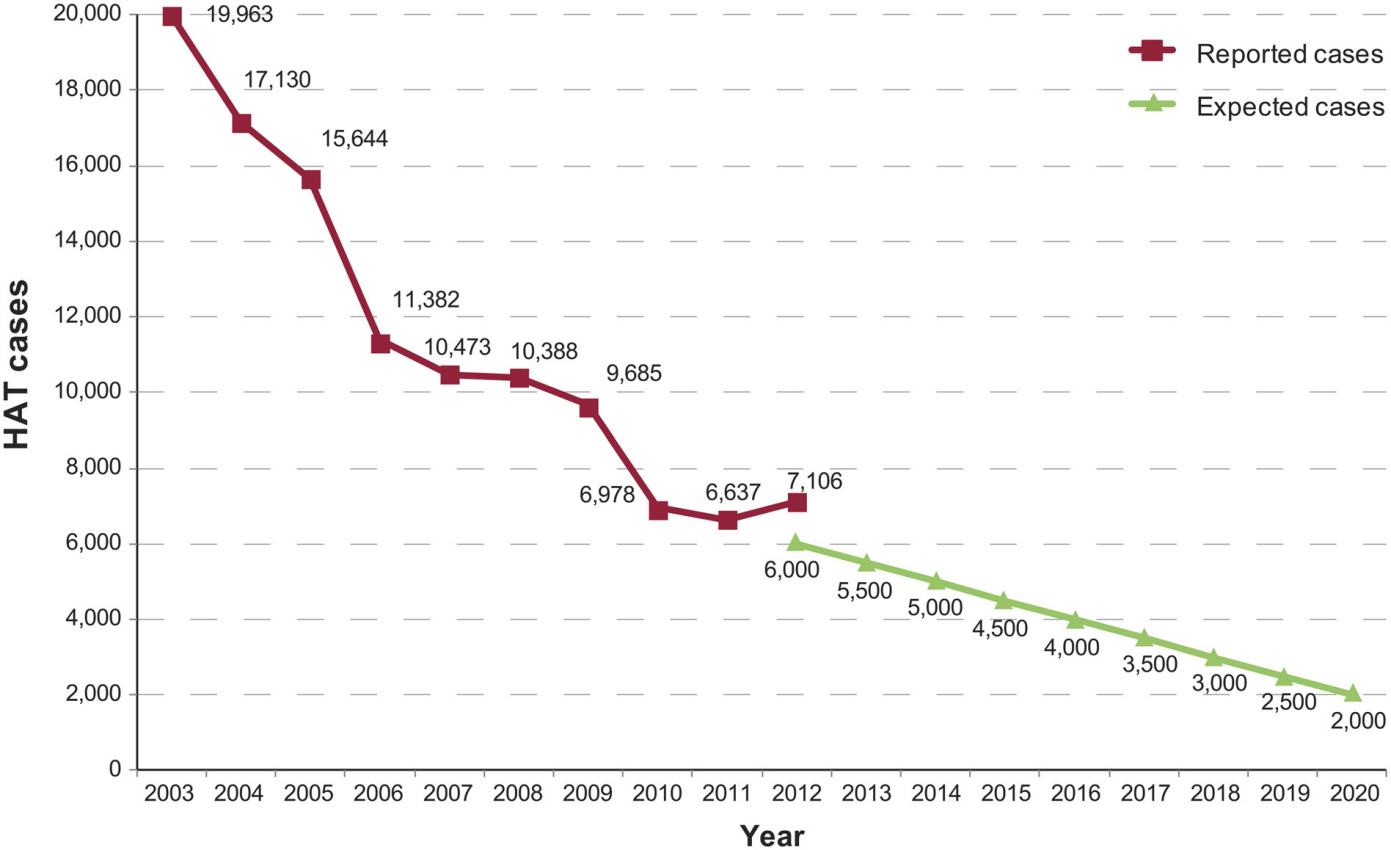
Gambiense HAT reported cases as compared with the benchmark for elimination (expected cases).

The first primary indicator for g-HAT elimination (i.e. number of reported cases by year) is illustrated in Fig 1, which also shows the expected progression of the indicator according to the scenario of elimination as a public health problem by 2020 [3]. A general decreasing trend was observed over the years. In 2012, an 18% excess of reported cases over the expected value was observed. That year, the renewed active screenings in the foci of Oriental province (DRC) and Ouham (Central African Republic) contributed to this surplus. In these areas, security constraints had prevented access for a few years, leading to detect infections in 2012 which had cumulated over the previous years when appropriate surveillance was lacking.

### Trends in the geographic distribution of gambiense HAT

**Fig 2:**
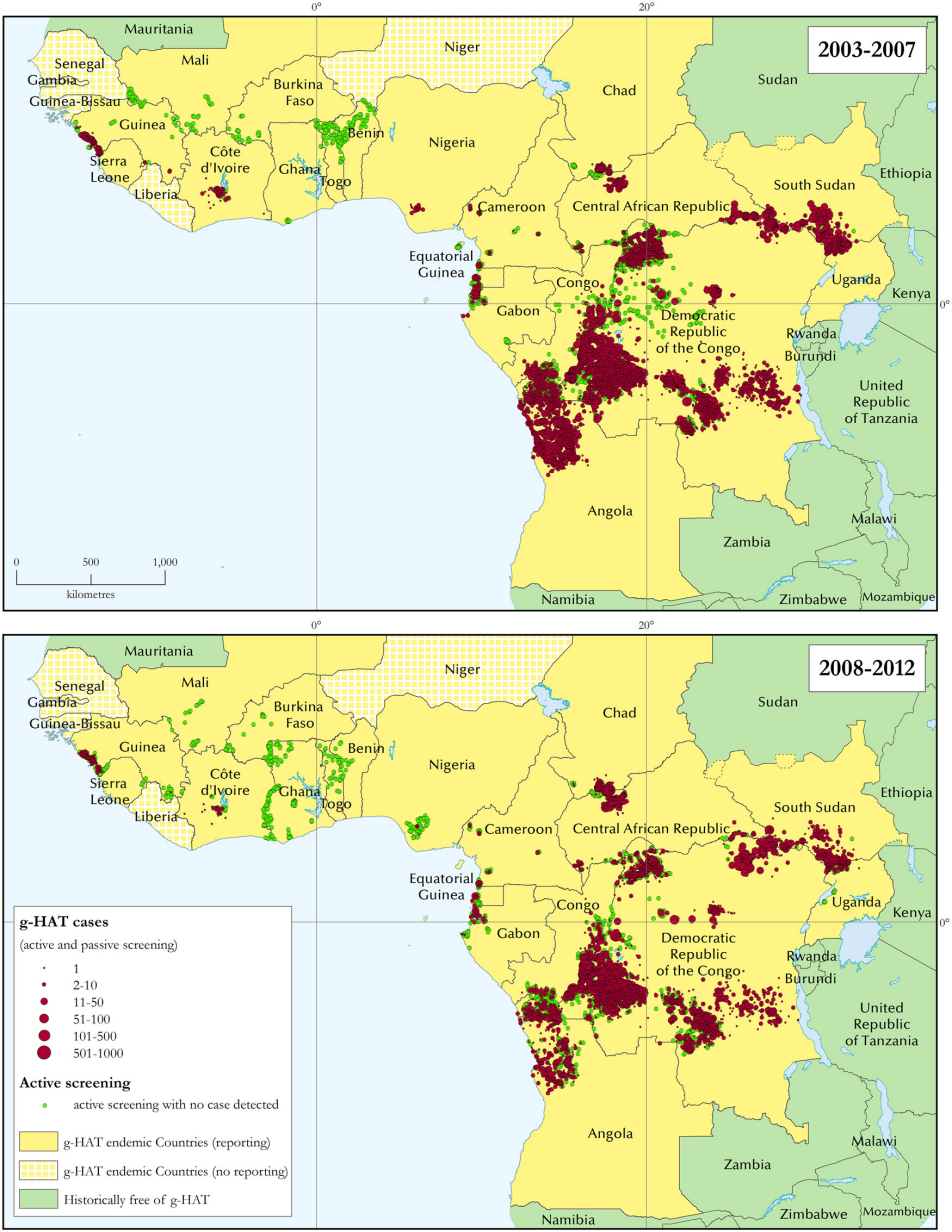
The distribution of gambiense human African trypanosomiasis. Periods 2003–2007 and 2008–2012.

The geographic extent of the disease has been identified as one of the secondary indicators of g-HAT elimination. G-HAT distribution maps are shown separately for the periods 2003–2007 and 2008–2012 (Fig 2). Regional distribution maps for West and Central Africa for the same periods are provided in S1 File.

A detailed status of mapping of g-HAT reported cases and geographical locations, as well as mapping accuracy, is presented in S2 File. In summary, 92% of g-HAT cases reported between 2003 and 2012 were localized at the village level, and 88% of the locations of epidemiological interest were geo-referenced. Using a methodology previously described [6], the average mapping accuracy for g-HAT cases is estimated at ≈ 1,000 m.

Looking at West Africa, in Guinea disease transmission has not declined significantly (436 reported cases in 2003–2007 versus 364 in 2008–2012), despite sustained active case-finding surveys in the costal foci characterized by a mangrove biotope. In response to this stagnant situation, in December 2011 vector control activities have been initiated in these areas with a view to complementing and synergizing with medical surveys. In Côte d'Ivoire, a decrease in the number of cases has been recorded (226 in 2003–2007 versus 49 in 2008–2012). Reduced active surveillance in the country due to civil unrest may have resulted in under-detection, thus showing this declining trend. However, when active case finding was resumed in 2009, no increase in the number of cases was noted. Active screening surveys targeted at all foci of Côte d'Ivoire are in the pipeline, aiming to improve our knowledge of the disease status. A similar drop in the number of g-HAT reported cases has been observed in Nigeria (65 in 2003–2007 versus 7 in 2008–2012), but a lack of sufficiently extensive passive and active screening activities should be taken into account.

Active case-finding surveys have continued to detect no cases in such countries as Burkina Faso, Benin, Ghana, Mali and Togo, thus underlining how mobile teams no longer offer a cost-effective approach to g-HAT surveillance in such epidemiological settings. Consequently, integrated passive surveillance in the health system has been initiated in 2010.

In Central Africa, social stability facilitated access to g-HAT foci in Cameroon, Congo, Equatorial Guinea and Gabon, where sustained active screening activities were carried out. In these countries, a significant and reliable decrease in the number of cases was observed (2,831 in 2003–2007 versus 646 in 2008–2012). In Chad, despite regular active screening in the most active focus (i.e. Mandoul), the number of reported cases did not show the expected decrease (1,268 in 2003–2007 versus 1,411 in 2008–2012). More systematic and extensive vector control to complement and synergize with medical surveys started in November 2013. In Central African Republic, reported cases (3,057 in 2003–2007 versus 3,156 in 2008–2012) are unlikely to reflect the reality of transmission on the ground because logistic and security constraints hamper access to the foci in Haut Mbomou and Ouham Prefectures. In South Sudan and Uganda a substantial decrease in the number of cases was reported (from 7,914 to 1,784 and from 1,616 to 462 respectively between 2003–2007 and 2008–2012). In Uganda the decrease was accompanied by extensive active and passive screening activities, while in South Sudan surveillance needs to be reinforced to ascertain the situation of disease transmission [13]. In Angola, the reported decrease in the number of cases was important (from 8,875 in 2003–2007 to 1,199 in 2008–2012), although there was also a slowdown in active case-finding activities, while maintaining passive screening capacities. Therefore, although an assessment in some areas could be required, there is a sense that the figures reported from Angola do reflect a real abatement. The Democratic Republic of the Congo (DRC) also displayed a marked decrease in the number of reported cases (from 48,304 in 2003–2007 to 31,716 in 2008–2012), while remaining the country burdened by the vast majority of g-HAT cases.

### Trends of gambiense HAT risk

**Fig 3:**
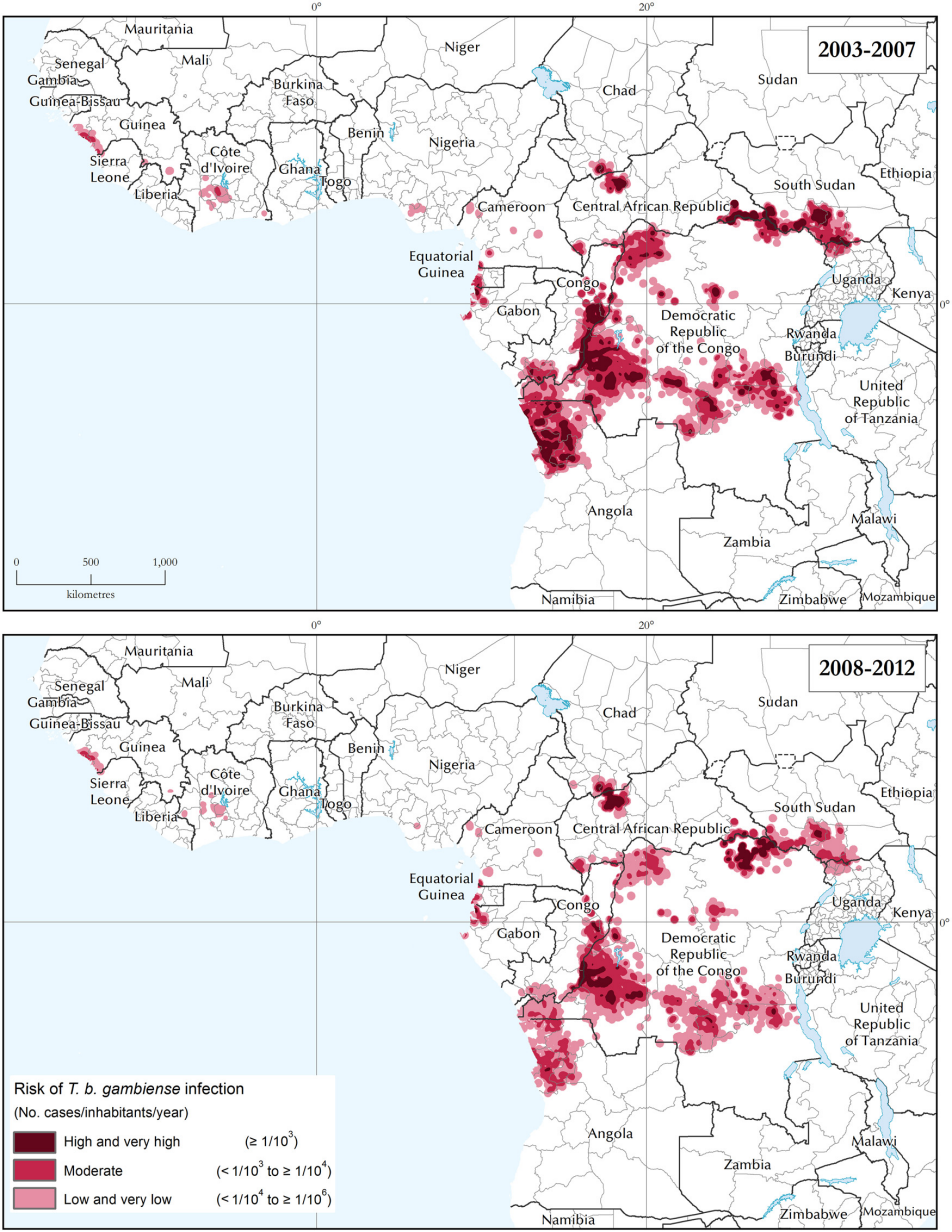
The distribution of the risk of *T*. *b*. *gambiense* infection. Periods 2003–2007 and 2008–2012.

**Fig 4:**
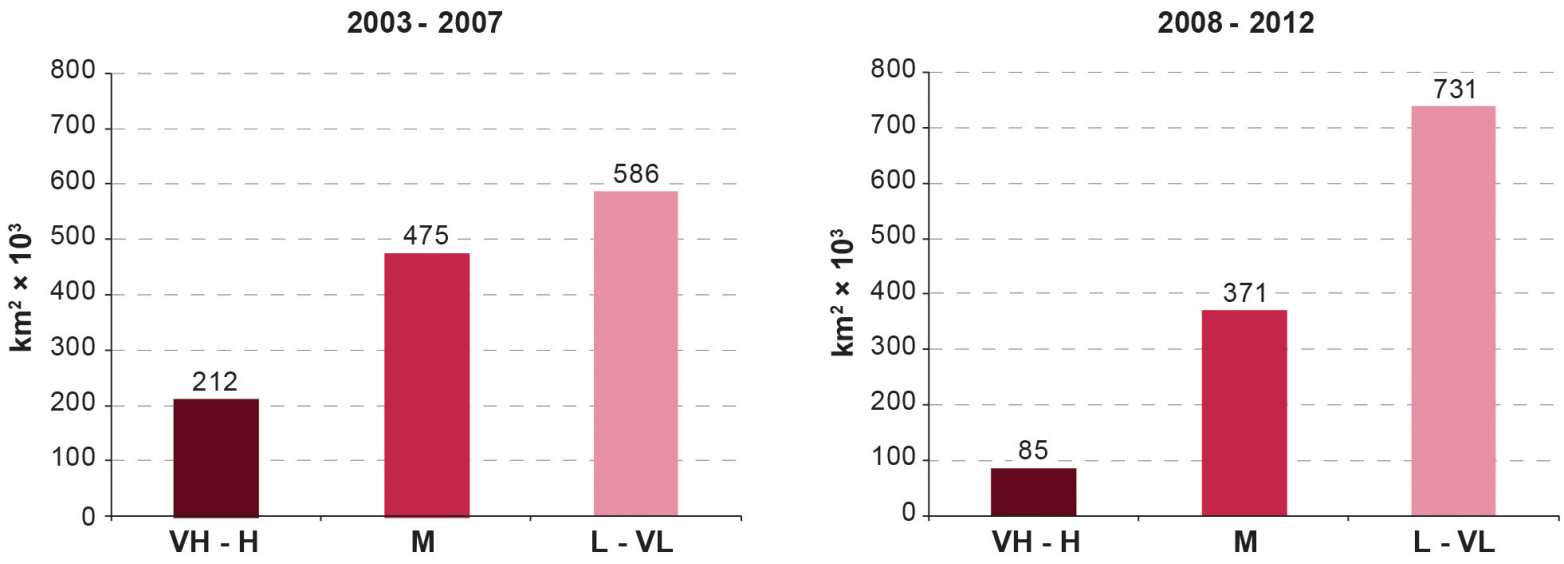
The areas at risk of *T*. *b*. *gambiense* infection (km^2^ × 10^3^). Periods 2003–2007 and 2008–2012. VH: Very High; H: High; M: Moderate; L: Low and VL: Very Low.

The area and population at different levels of risk represent an additional secondary indicator to assess the impact of the elimination activities. With regard to areas at different levels of risk, the progress between the two 5-year study periods (2003–2007 and 2008–2012) is mapped in Fig 3, summarized at continental level in Fig 4, and presented on a country-by-country basis in Table 1. Regional risk maps for West and Central Africa are provided in S3 File.

**Table 1:** Areas at risk of *T*. *b*. *gambiense* infection (km^2^).

Country	Total country area*	Area at risk 2003–2007					Area at risk 2008–2012				
		Very High and High	Moderate	Low and Very Low	Total at risk	% of total country area	Very High and High	Moderate	Low and Very Low	Total at risk	% of total country area
Angola	1,253,770	51,716	67,363	57,370	176,449	14.1	637	51,134	86,531	138,301	11.0
Cameroon	466,396	-	2,057	14,760	16,817	3.6	-	1,381	13,418	14,799	3.2
Central African Republic	624,398	18,749	21,318	19,066	59,133	9.5	13,072	22,556	30,290	65,918	10.6
Chad	1,272,490	2,237	3,393	7,553	13,183	1.0	3,143	3,960	12,665	19,768	1.6
Congo	338,522	22,779	41,447	44,591	108,817	32.1	6,132	23,152	42,682	71,966	21.3
Côte d’Ivoire	321,363	-	2,053	20,332	22,385	7.0	-	-	15,104	15,104	4.7
Democratic Republic of the Congo	2,304,080	85,788	275,294	356,739	717,821	31.2	58,367	222,111	434,392	714,870	31.0
Equatorial Guinea	27,019	198	4,053	2,273	6,523	24.1	-	1,901	2,347	4,248	15.7
Gabon	265,978	1,069	5,730	6,504	13,303	5.0	515	5,674	7,305	13,494	5.1
Guinea	246,094	59	4,239	14,494	18,793	7.6	-	3,378	9,469	12,847	5.2
Liberia	96,480	-	-	11	11	0.0	-	-	-	-	-
Nigeria	908,866	-	-	7,595	7,595	0.8	-	-	1,788	1,788	0.2
Sierra Leone	72,777	-	2	1,651	1,653	2.3	-	-	1,069	1,069	1.5
South Sudan	633,356	27,428	40,489	26,971	94,889	15.0	2,956	33,321	59,442	95,719	15.1
Uganda	205,540	1,465	7,976	6,449	15,890	7.7	1	2,302	14,442	16,745	8.1
Other Endemic Countries**	3,366,570	-	-	-	-	-	-	-	-	-	-
Total	12,553,313	211,489	475,412	586,360	1,273,261	10.1	84,822	370,871	730,943	1,186,636	9.5

Periods 2003–2007 and 2008–2012.

* Land area. The area of surface water bodies as depicted in the Shuttle Radar Topography Mission—River-Surface Water Bodies dataset [28] is not included.

** Countries at marginal risk: Benin, Burkina Faso, Gambia, Ghana, Guinea-Bissau, Mali, Niger, Senegal and Togo.

We note that, between the two periods, the total area at high or very high risk of g-HAT was reduced by 60% (from 211 to 85 thousand km^2^), while the area at moderate risk decreased by 22% (from 475 to 371 thousand km^2^). The reduction in these categories resulted in an increase of 25% (from 586 to 731 thousand km^2^) in the area at low or very low risk. Overall, the area at risk of g-HAT shrank by 6% (from 1.27 to 1.19 million km^2^). A few salient risk patterns and trends can be highlighted at the national level. In Equatorial Guinea, Uganda and Angola we observe the virtual disappearance of the areas at high and very high risk. The reduction in these risk categories was of 89% in South Sudan, 73% in Congo, 30% in CAR, and 32% in DRC, which by itself accounts for over half of the total risk area. Only Chad has experienced an increase of area at high and very high risk.

**Fig 5:**
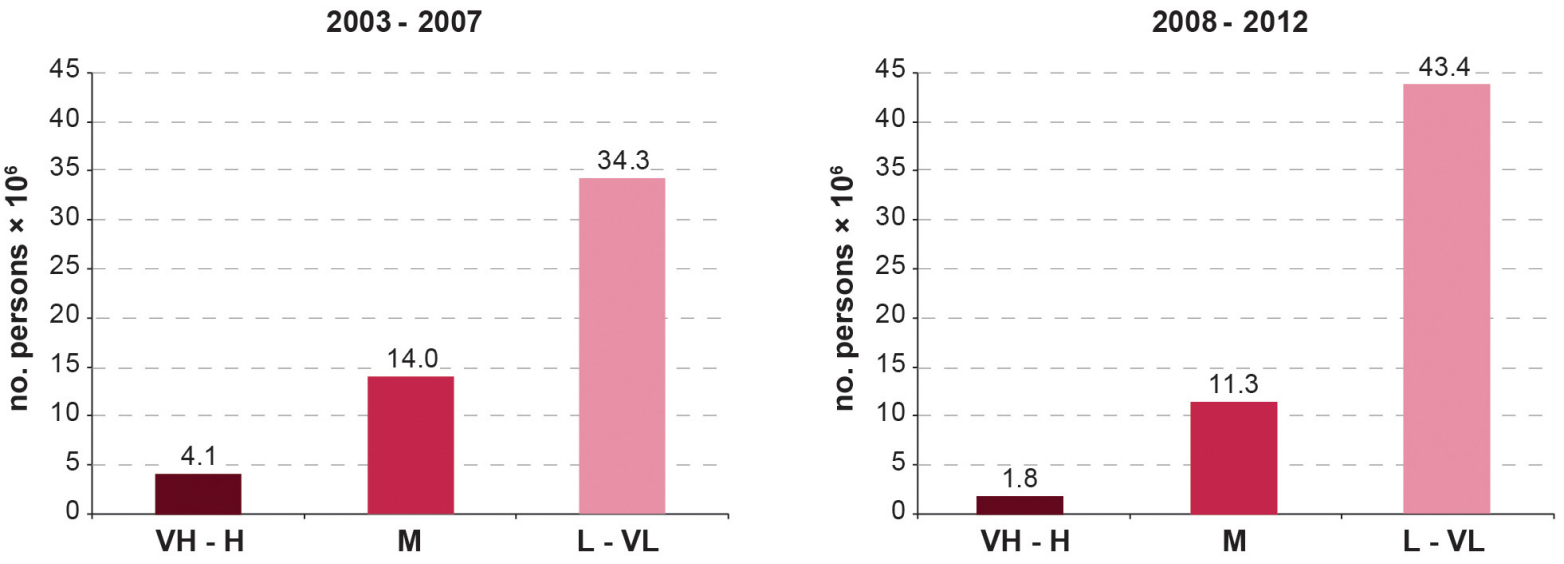
The population at risk of *T*. *b*. *gambiense* infection (no. persons × 10^6^). Periods 2003–2007 and 2008–2012. VH: Very High; H: High; M: Moderate; L: Low and VL: Very Low.

The pattern observed for the areas at risk is mirrored by the population at risk. Fig 5 summarizes the trends at continental level of the total population at different levels of g-HAT risk, while Table 2 provides country-level details comparing the two 5-year study periods (2003–2007 and 2008–2012). It is observed a 57% decrease for the categories very high and high (from 4.1 million to 1.8 million) and a 20% decrease for the population at moderate risk (from 14.0 million to 11.3 million people). People living at low or very low risk of g-HAT have increased by 27% (from 34.3 million to 43.4 million). The latter increase is not only due to the passage of certain populations from higher to lower risk status, but also to population growth. The net effect of these combined factors is an increase in the total population at risk from 52.4 to 56.4 million. Nevertheless, it is noteworthy that according to the criteria defined by the WHO Expert Committee on HAT [4], over three quarters of the total population at risk of g-HAT (i.e. 43.4 million) have reached a status where the disease is no longer considered a public health problem (i.e. low and very low risk, i.e. < 1 case per 10,000 inhabitants p.a.) [4].

**Table 2:** Population at risk of *T*. *b*. *gambiense* infection (no. persons).

Country	Total country population 2007*	Population at risk 2003–2007					Total country population 2012**	Population at risk 2008–2012				
		Very High and High	Moderate	Low and Very Low	Total at risk	% of total country population		Very High and High	Moderate	Low and Very Low	Total at risk	% of total country population
Angola	12,263,600	618,453	868,986	3,003,536	4,490,975	36.6	18,056,072	3,653	946,337	4,902,960	5,852,950	32.4
Cameroon	18,060,400	-	23,251	617,822	641,073	3.5	20,129,878	-	23,079	372,837	395,916	2.0
Central African Republic	4,369,030	60,094	137,323	217,745	415,162	9.5	5,057,208	79,845	187,710	295,294	562,849	11.1
Chad	9,885,660	73,995	118,061	236,314	428,370	4.3	10,975,648	96,949	175,032	455,673	727,654	6.6
Congo	3,800,620	90,372	452,514	1,852,444	2,395,330	63.0	4,366,266	16,058	121,195	2,336,968	2,474,221	56.7
Côte d’Ivoire	18,013,400	-	175,240	1,711,429	1,886,669	10.5	21,952,093	-	-	1,457,324	1,457,324	6.6
Democratic Republic of the Congo	65,751,500	2,774,564	10,535,300	19,803,560	33,113,424	50.4	73,599,190	1,536,059	8,264,390	26,768,590	36,569,039	49.7
Equatorial Guinea	551,201	828	22,390	19,230	42,448	7.7	685,991	-	19,257	10,901	30,158	4.4
Gabon	1,454,870	3,621	18,121	741,891	763,633	52.5	1,608,321	2,261	24,378	821,399	848,038	52.7
Guinea	9,947,820	5	192,720	2,512,548	2,705,273	27.2	10,884,958	-	163,604	2,157,291	2,320,895	21.3
Liberia	3,195,930	-	-	1,954	1,954	0.1	3,887,886	-	-	-	-	-
Nigeria	135,031,000	-	-	2,316,446	2,316,446	1.7	170,123,740	-	-	467,946	467,946	0.3
Sierra Leone	6,144,560	-	57	206,612	206,669	3.4	5,485,998	-	-	109,597	109,597	2.0
South Sudan	6,815,170	366,167	488,844	319,063	1,174,074	17.2	10,625,176	41,711	1,132,700	1,201,054	2,375,465	22.4
Uganda	30,262,600	143,911	1,001,330	722,190	1,867,431	6.2	34,640,833	8	212,196	2,006,355	2,218,559	6.4
Other Endemic Countries***	91,611,040	-	-	-	-	-	106,765,169	-	-	-	-	-
Total	417,158,401	4,132,010	14,034,137	34,282,784	52,448,931	12.6	498,844,427	1,776,544	11,269,878	43,364,189	56,410,611	11.3

Periods 2003–2007 and 2008–2012

* As per Landscan 2007

** As per Landscan 2012

*** Countries at marginal risk: Benin, Burkina Faso, Gambia, Ghana, Guinea-Bissau, Mali, Niger, Senegal and Togo.

CAR and Chad appear to be the only notable exceptions to this declining pattern. CAR shows an increase of 33% for the categories very high and high risk, whereas Chad shows a 31% increase, albeit over a relatively circumscribed area (i.e. 3,100 km^2^).

## Discussion

The number of new cases reported by year is the first primary indicator to monitor the elimination of g-HAT. The data presented in this paper show for 2012 an excess of 1,106 cases as compared to the target. When looking at this indicator it is important to stress that small fluctuations in the number of reported cases may reflect circumstances of surveillance rather than epidemiological trends. Therefore, great caution must be taken when interpreting trends in reported cases, especially when unexpected increases or decreases occur. The steadily decreasing trend resumed in 2013 [14], with a narrowing of the gap between reported cases and the target (i.e. an excess of 728 cases). Discussing in detail the reasons for the observed decrease in the number of cases is beyond the scope of this paper. Suffice it say that the results were achieved through outstanding efforts in disease surveillance and control by NSSCP, with the support of international organizations led by WHO and the financial assistance of the private sector and of major international donors [15].

The database of the Atlas of HAT is progressively improving in completeness and accuracy, thus enhancing its reliability. The Atlas represents a key tool to follow the geographic distribution of the disease, and to monitor g-HAT control and elimination.

The robustness of the Atlas of HAT enables the estimation of populations and areas at risk, which in turn provides information on two secondary indicators that assess the intensity and effectiveness of the elimination activities (i.e. (i) the geographical extent of the disease and (ii) the populations at different levels of risk). All risk estimations presented in this paper are solely based on g-HAT cases detected and reported from field actors involved in control and surveillance. As such, the estimates are affected by under-detection and underreporting at a degree that is presently difficult to quantify. To address this gap, a spatial modelling framework is being developed. This modelling exercise is expected to shed some light into the magnitude and distribution of under-detection. Arguably, as g-HAT elimination progresses and reported cases decrease, efforts will have to be made to improve our knowledge of the factors that may influence the risk of disease re-emergence and re-introduction. Modelling exercises could contribute to shed some light on these factors.

Gaps and limitations notwithstanding, there is sufficient evidence to support the notion of a real overall decline of transmission of g-HAT. In West Africa, the combined effect of public health interventions and climatic and demographic changes [16] have provoked a dramatic decline of disease transmission rendering active case-finding surveys no longer cost effective in the majority of the foci. As a first step to respond to this epidemiological status of the disease, in 2010 a control and surveillance strategy integrated in the health system was piloted in Benin and Togo, followed in 2014 by Côte d’Ivoire, Ghana and Guinea. Burkina Faso, Guinea Bissau, Mali, Niger, Senegal and Sierra Leone are planned for 2015.

A similar decline in transmission is apparent in Central Africa, with the exception of areas affected by insecurity such as the foci in northern CAR—connected to the foci in southern Chad—as well as the Oriental province in DRC. In DRC logistical hurdles compound security constraints in making it difficult to draw an accurate picture of the extent of the disease in some parts of the country. Overall, it would appear that the shrinking number of reported cases in DRC reflect a real decline in disease transmission. This notion is corroborated by the increasing difficulties in recruiting patients for clinical trials [17]. In this epidemiological context, as of 2014 a control and surveillance strategy integrated in the health system has been already deployed in Cameroon, Chad, Congo, Equatorial Guinea, Gabon and Uganda, while DRC and South Sudan are planned for 2015.

Concerning the population at risk, the challenge for the future is twofold. First, to prevent the 43.4 million people presently living in low and very low risk areas from sliding back into a situation of higher risk through effective surveillance and response. Second, to setup appropriate and sustainable control strategies to reduce transmission in the areas where 13 million of people are still living at moderate to very high risk of infection. If met, these targets will enable to reach the 2020 goal of eliminating g-HAT as a public health problem [2]. In the process, additional steps will have to be planned to reach the complete interruption of transmission by 2030 [3].

The availability of new control tools, i.e. individual screening test [18,19], oral treatments [20,21], and simpler and cheaper devices for vector control [22] will facilitate sustainable control and surveillance based on the involvement of the regular health system, including the peripheral level. Technical advances are coupled with a positive political momentum [23,24] and the commitment of strong stakeholders [25]. At the same time, risk factors still exist that could jeopardize the achievement of the set goals; these include possible donor fatigue, competing health priorities at the national level, social unrest [26], gaps in the coverage of population at risk [5], challenges in the progressive integration of vertical programmes into often weak health systems, and dwindling resources for a still needed operational research [4]. Also, to tackle the risk of duplication of efforts and competition for prominence that can jeopardize the process, a coordinated and unequivocal support of g-HAT stakeholders to disease endemic countries must be articulated. In this regard, the WHO network for the elimination of HAT, set up in March 2014, provides an opportunity and a tool to synergize efforts and to overcome, in a coordinated manner, the expected and unexpected obstacles we are bound to face in this exciting and challenging endeavour [15]. As already stated [27], the journey towards elimination of g-HAT is not far, nor easy.

## Supporting Information

S1 File The distribution of gambiense HAT in Western and Central Africa. (DOCX)

S2 File Progress status and accuracy of mapping for g-HAT cases and geographic locations (Period 2003–2012). (DOCX)

S3 File The risk of gambiense HAT in Western and Central Africa. (DOCX)
