# Supplementary material for: Monitoring the Progress towards the Elimination of Gambiense Human African Trypanosomiasis
Source: PLoS Negl Trop Dis. 2015 Jun 9;9(6):e0003785. doi: 10.1371/journal.pntd.0003785 (PMC4461311; doi:10.1371/journal.pntd.0003785)
Supplement: S2 File — (DOCX) [file pntd.0003785.s002.docx]

# Progress status and accuracy of mapping for g-HAT cases and geographic locations (Period 2003 – 2012)

## Progress status of mapping of g-HAT cases and geographic locations

Table 1 Progress status of mapping of g-HAT cases and geographic locations (period 2003-2012)

| **Country** | **HAT cases** | | **Geographic locations** | |
| --- | --- | --- | --- | --- |
|  | **Reported (number)** | **Mapped (%)** | **Reported (number)** | **Mapped (%)** |
| Angola | 10,074 | 91.9 | 3,170 | 82.1 |
| Benin | 0 | - | 123 | 100.0 |
| Burkina Faso | 0 | - | 63 | 100.0 |
| Cameroon | 150 | 100.0 | 100 | 100.0 |
| Central African Republic | 6,213 | 97.2 | 583 | 99.3 |
| Chad | 2,679 | 86.3 | 271 | 94.8 |
| Congo | 2,933 | 97.8 | 891 | 99.0 |
| Côte d’Ivoire | 275 | 92.4 | 164 | 98.2 |
| Democratic Republic of the Congo | 80,020 | 92.9 | 17,308 | 86.5 |
| Equatorial Guinea | 119 | 100.0 | 120 | 100.0 |
| Gabon | 275 | 100.0 | 151 | 100.0 |
| Ghana | 0 | - | 160 | 98.8 |
| Guinea | 800 | 99.8 | 435 | 98.6 |
| Mali | 0 | - | 97 | 83.5 |
| Nigeria | 72 | 100.0 | 95 | 97.9 |
| Sierra Leone | 0 | - | 24 | 100.0 |
| South Sudan | 9,698 | 78.6 | 719 | 89.6 |
| Togo | 0 | - | 84 | 100.0 |
| Uganda | 2,078 | 100.0 | 856 | 97.7 |
| TOTAL | 115,386 | 92.1 | 25,414 | 88.0 |

## Geographical accuracy of mapped villages

Various sources of geographic information are used to build the Atlas of HAT, and they are characterized by different levels of accuracy. To estimate the overall spatial accuracy of the resulting maps, locations are classified into four broad categories of accuracy: very high, high, moderate and low (Simarro et al. 2010).

The category “very high accuracy” includes locations whose coordinates were derived from GPS measurements carried out by field teams in charge of HAT reporting. We estimate an average error of 50 m for these coordinates. Certain coordinates obtained from gazetteers are also included in the first category of accuracy, but only if no rounding of the coordinates was carried out (i.e. coordinates must be available from gazetteers in full, as degrees, minutes and seconds, or down to the 4^th^ decimal place if decimal degrees notation was used).

The category “high accuracy” includes all those locations where coordinates obtained from gazetteers have, for some reason, been rounded (or truncated) at the source. For example, in the GEOnet Names Server database^[[1]](#footnote-1)^, a sizable proportion of the coordinates for Africa are rounded to the nearest minute, which results in errors of up to 928 m. For this second category we therefore estimated an average error of 500 m.

“Medium” and “low” accuracy refer to coordinates that were estimated by using digital and paper maps, out-of-scale sketches of survey areas, as well as qualitative information received from field-workers. We estimate an average accuracy of 2,500 m and 5,000 m for the categories “medium” and “low” respectively, depending on the detail of the qualitative information they are based upon.

The summary of mapping accuracy for villages and for g-HAT cases is provided in Table 2 and Table 3 respectively.

Table 2 Geographical accuracy for mapped villages (period 2003-2012)

| **Country** | **Category of accuracy for mapped villages (number of villages)** | | | | **Average accuracy (m)** |
| --- | --- | --- | --- | --- | --- |
|  | **Very high** | **High** | **Moderate** | **Low** |  |
| Angola | 1,478 | 578 | 204 | 341 | 1,000 |
| Benin | 68 | 36 | 17 | 2 | 600 |
| Burkina Faso | 56 | 2 | 1 | 4 | 400 |
| Cameroon | 66 | 19 | 15 | 0 | 500 |
| Central African Republic | 307 | 102 | 98 | 72 | 1,200 |
| Chad | 185 | 36 | 25 | 11 | 600 |
| Congo | 508 | 224 | 85 | 65 | 800 |
| Côte d'Ivoire | 105 | 32 | 16 | 8 | 600 |
| Democratic Republic of the Congo | 5,862 | 2,383 | 4,839 | 2,081 | 1,600 |
| Equatorial Guinea | 71 | 20 | 28 | 1 | 700 |
| Gabon | 47 | 61 | 31 | 12 | 1,100 |
| Ghana | 145 | 13 | 0 | 0 | 100 |
| Guinea | 331 | 36 | 57 | 5 | 500 |
| Mali | 71 | 10 | 0 | 0 | 100 |
| Nigeria | 66 | 15 | 10 | 2 | 500 |
| Sierra Leone | 24 | 0 | 0 | 0 | 100 |
| South Sudan | 167 | 70 | 323 | 84 | 2,000 |
| Togo | 76 | 6 | 2 | 0 | 100 |
| Uganda | 620 | 29 | 127 | 60 | 800 |
| Total | 11,232  (43.9%) | 4,160  (16.3%) | 7,024  (27.4%) | 3,178  (12.4%) | 1,400 |

Table 3 Geographical accuracy for g-HAT mapped cases (period 2003-2012)

| **Country** | **Category of accuracy for g-HAT mapped cases (number of g-HAT cases)** | | | | **Average accuracy (m)** |
| --- | --- | --- | --- | --- | --- |
|  | **Very high** | **High** | **Moderate** | **Low** |  |
| Angola | 5,236 | 1,872 | 777 | 1,368 | 526 |
| Benin | 0 | 0 | 0 | 0 | - |
| Burkina Faso | 0 | 0 | 0 | 0 | - |
| Cameroon | 116 | 29 | 5 | 0 | 35 |
| Central African Republic | 4,929 | 523 | 240 | 349 | 228 |
| Chad | 2,041 | 121 | 136 | 15 | 172 |
| Congo | 2,320 | 396 | 105 | 48 | 135 |
| Côte d'Ivoire | 188 | 44 | 14 | 8 | 377 |
| Democratic Republic of the Congo | 40,808 | 8,814 | 14,478 | 10,208 | 1,239 |
| Equatorial Guinea | 63 | 17 | 39 | 0 | 46 |
| Gabon | 152 | 110 | 12 | 1 | 167 |
| Ghana | 0 | 0 | 0 | 0 | - |
| Guinea | 751 | 29 | 17 | 1 | 93 |
| Mali | 0 | 0 | 0 | 0 | - |
| Nigeria | 37 | 6 | 28 | 1 | 427 |
| Sierra Leone | 0 | 0 | 0 | 0 | - |
| South Sudan | 2,911 | 1,236 | 2,971 | 509 | 1,033 |
| Togo | 0 | 0 | 0 | 0 | - |
| Uganda | 1,872 | 57 | 217 | 56 | 890 |
| Total | 61,424  (57.8%) | 13,254  (12.5%) | 19,039  (17.9%) | 12,564  (11.8%) | 1,027 |

## References

Simarro, P.P., Cecchi, G., Paone, M., Franco, J.R., Diarra, A., Ruiz, J.A., Fèvre, E.M., Courtin, F., Mattioli, R.C. and Jannin, J.G., 2010. The Atlas of human African trypanosomiasis: a contribution to global mapping of neglected tropical diseases, International Journal of Health Geographics, **9**, 57.

1. <http://earth-info.nga.mil/gns/html/> [↑](#footnote-ref-1)
