# Supplementary material for: Monitoring the Progress towards the Elimination of Gambiense Human African Trypanosomiasis
Source: PLoS Negl Trop Dis. 2015 Jun 9;9(6):e0003785. doi: 10.1371/journal.pntd.0003785 (PMC4461311; doi:10.1371/journal.pntd.0003785)
Supplement: S3 File — (DOCX) [file pntd.0003785.s003.docx]

# The risk of gambiense HAT in Western Africa


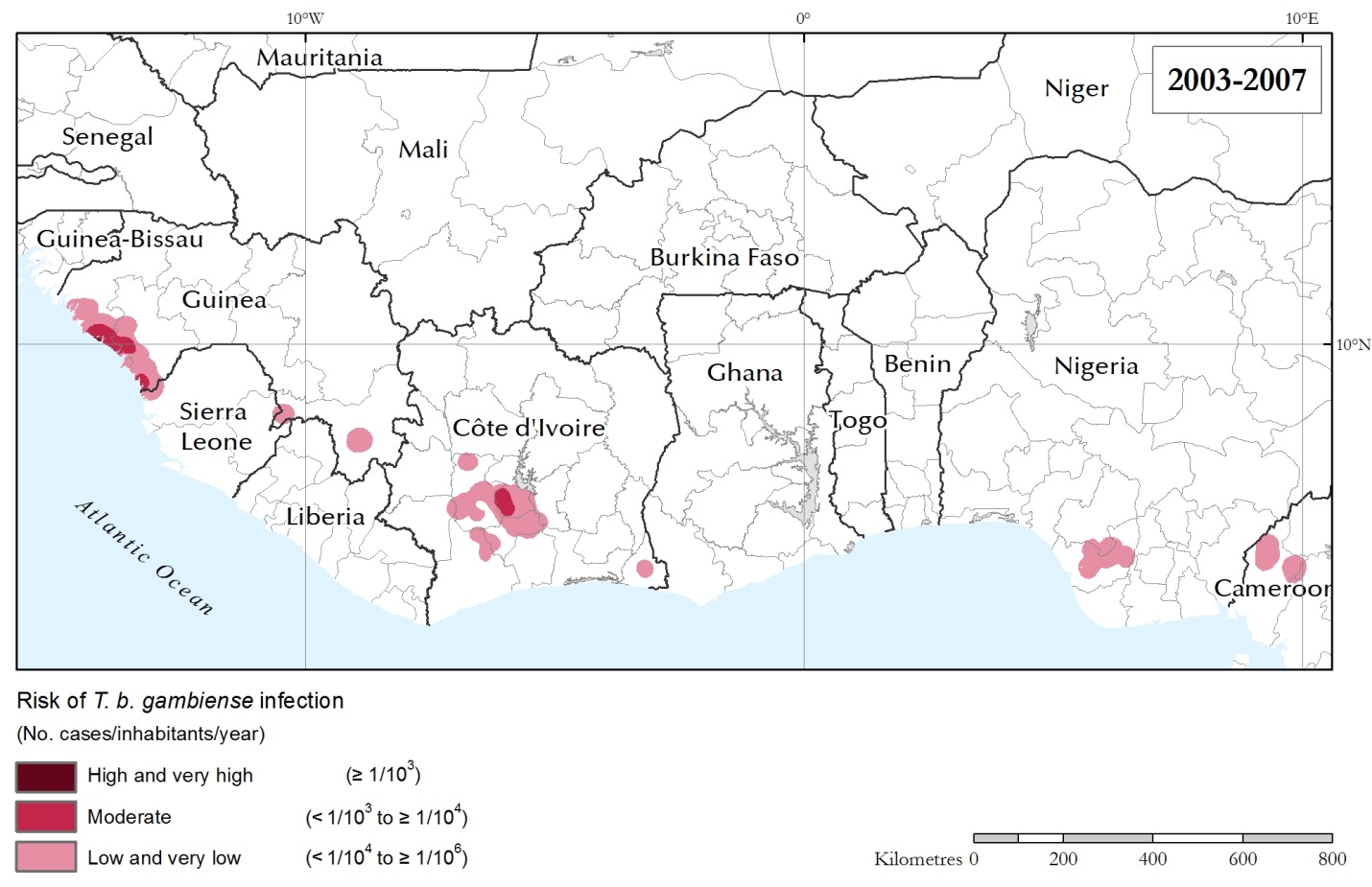


Figure 1 The risk of gambiense HAT in Western Africa (2003-2007)


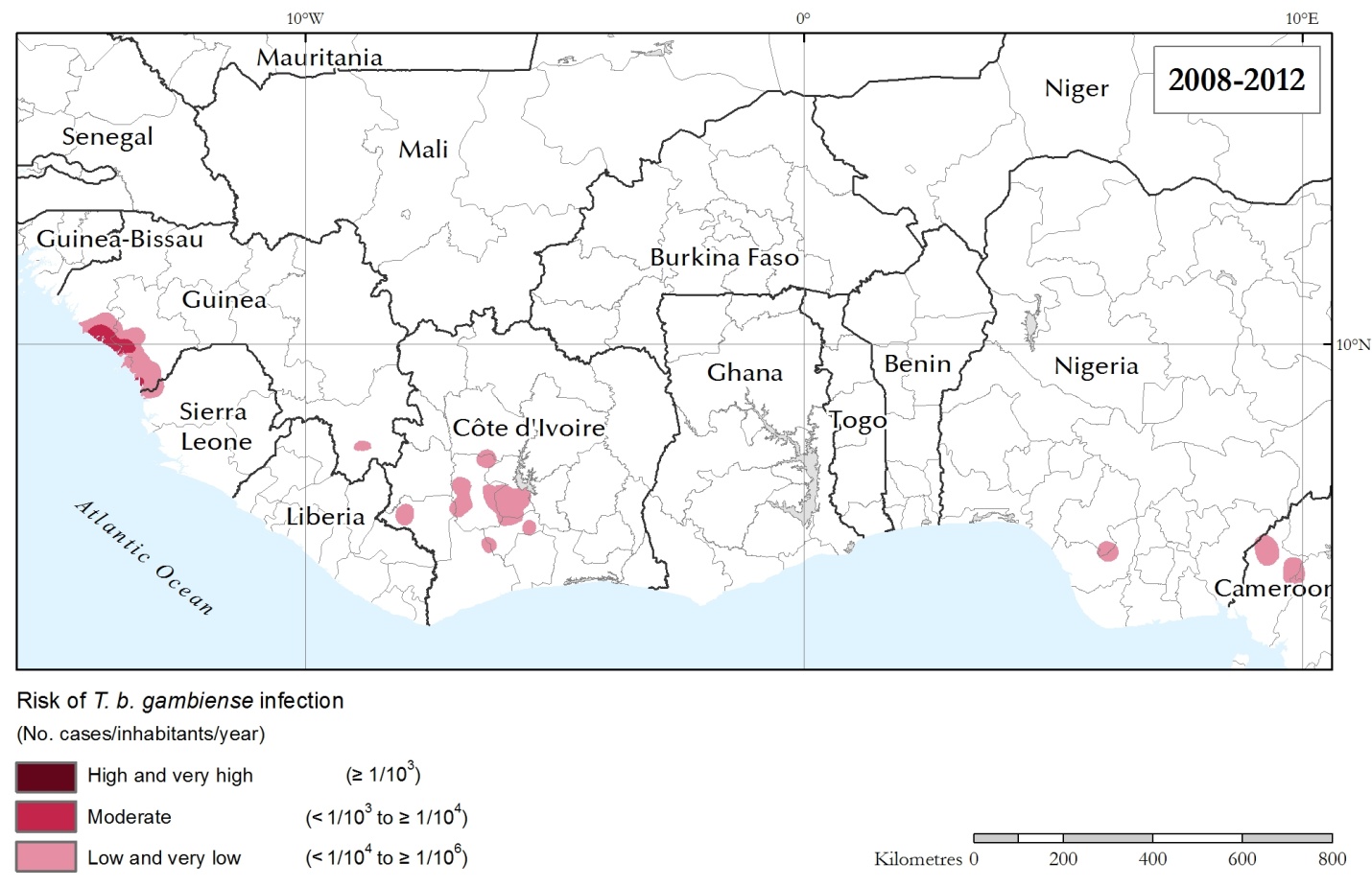


Figure 2 The risk of gambiense HAT in Western Africa (2008-2012)

# The risk of gambiense HAT in Central Africa


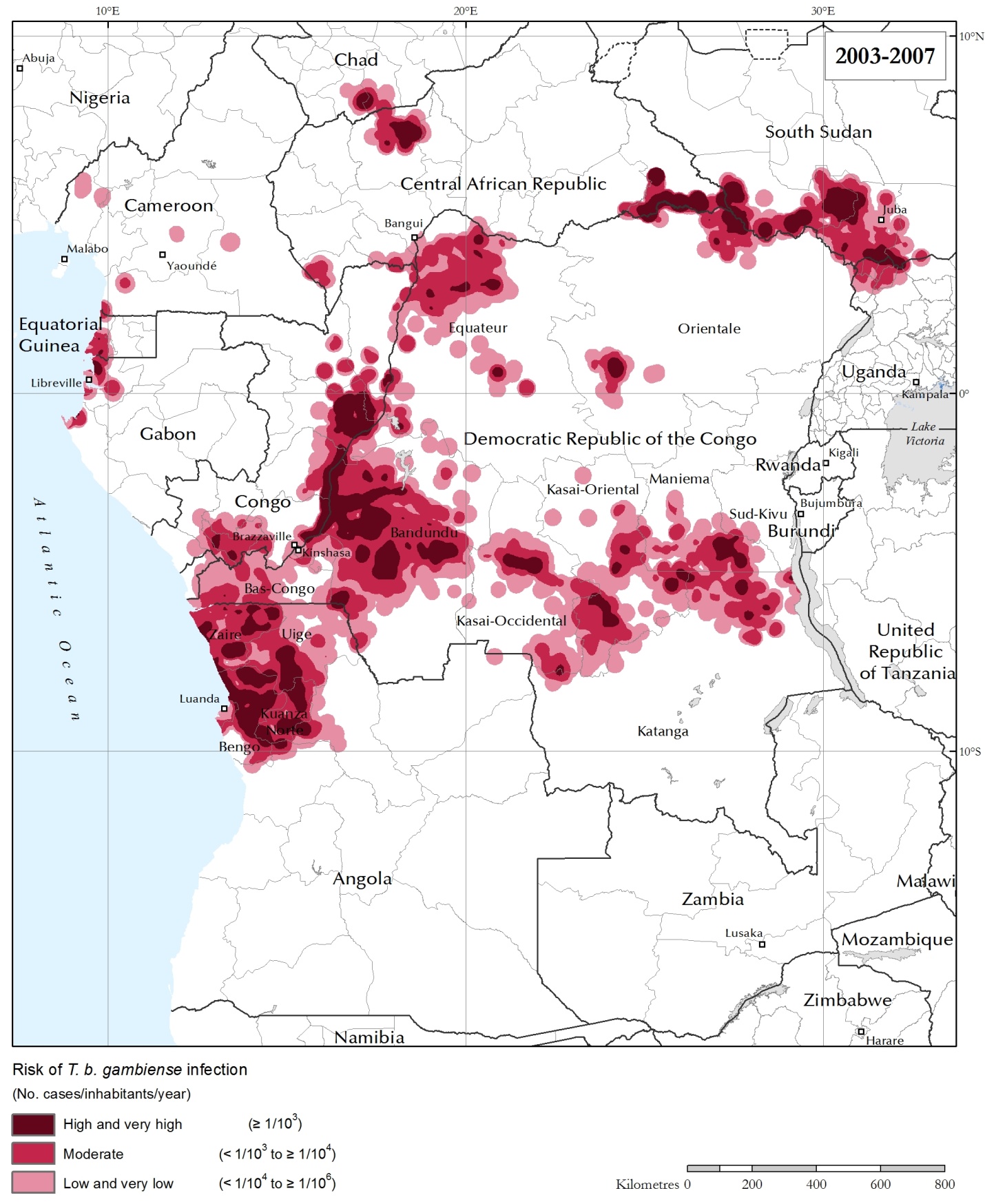


Figure 3 The risk of gambiense HAT in Central Africa (2003-2007)


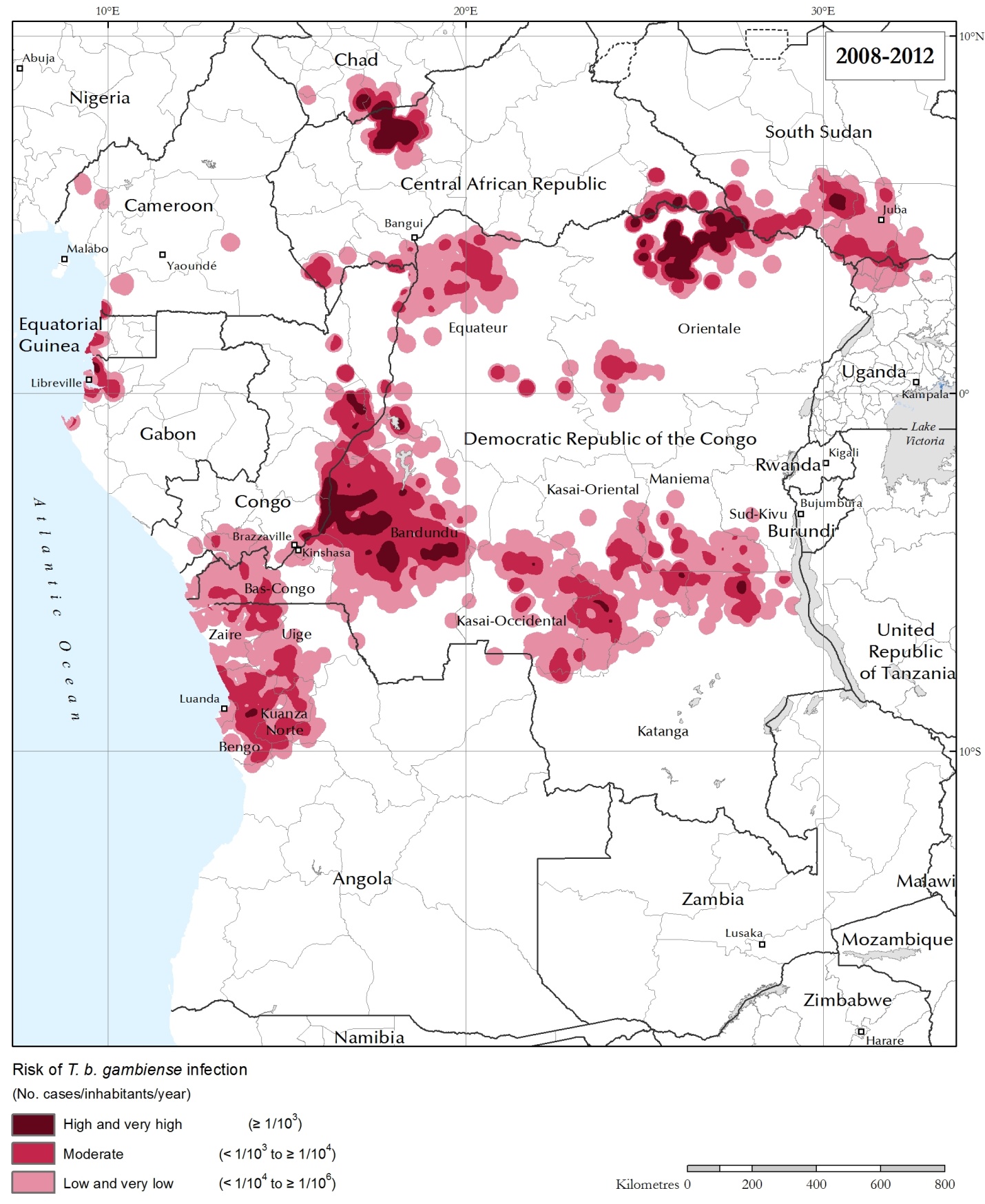


Figure 4 The risk of gambiense HAT in Central Africa (2008-2012)
